# The impact of dysphagia on quality of life in stroke patients

**DOI:** 10.1097/MD.0000000000021795

**Published:** 2020-08-21

**Authors:** Doo-Young Kim, Hyo-Sik Park, Si-Woon Park, Jae-Hyung Kim

**Affiliations:** a Department of Rehabilitation Medicine, Catholic Kwandong University International St. Mary's Hospital and Catholic Kwandong University, College of Medicine, Incheon; b Department of Rehabilitation Medicine, Eulji University Hospital and Eulji University School of Medicine, Daejeon, Korea.

**Keywords:** deglutition, deglutition disorder, fluoroscopy, quality of life, stroke

## Abstract

The objective of this study was to investigate the quality of life in stroke patients using a swallowing quality of life (SWAL-QOL) questionnaire. The correlation between SWAL-QOL questionnaire outcome and videofluoroscopic dysphagia scale (VDS) scores in stroke patients was also determined.

This cross-sectional study was retrospectively conducted with 75 stroke patients with dysphagia symptoms. Videofluoroscopic swallowing study (VFSS) and SWAL-QOL questionnaires were performed for all patients. These patients were divided into an oral feeding group and a tube feeding group. SWAL-QOL scores were compared between the 2 groups. The severity of dysphagia was estimated by VDS scores according to the videofluoroscopic swallowing study results. The relationships between SWAL-QOL scores and VDS scores were also investigated.

The composite score was 48.82 ± 19.51 for the tube feeding group and 53.17 ± 25.42 for the oral feeding group. There were significant differences in burden and sleep subdomains of the SWAL-QOL between the 2 groups (*P* = .005 and *P* = .012, respectively). There was a significant negative correlation between the composite score of SWAL-QOL outcome and the total VDS score (*r* = −0.468, *P* = .012). The pharyngeal-phase score of the VDS had significant negative correlations with the SWAL-QOL subdomains of burden (*r* = −0.327, *P* = .013), mental health (*r* = −0.348, *P* = .008), and social functioning (*r* = −0.365, *P* = .029).

To improve the quality of life of stroke patients, dysphagia rehabilitation should focus on the pharyngeal phase of dysphagia.

## 1 Introduction

Dysphagia is commonly found in acute stroke patients. The prevalence of dysphagia ranges from 50% to 80%.^[1–5]^ Patients with dysphagia could recover within several weeks. However, prolonged dysphagia can cause severe comorbidities, such as pneumonia, dehydration, malnutrition, and even death. It adversely affects patients’ quality of life and mental health.^[6–8]^ And dietary modification are required. If severe, the patients must be fed with tubes, such as a nasogastric tube or a percutaneous endoscopic gastrostomy tube.^[9]^

Most treatments for dysphagia have been focused on improving physiologic parameters in a swallowing study. However, a swallowing dysfunction influences not only physiologic aspects, but also social and psychologic aspects.^[10]^ In 2000, the swallowing quality of life (SWAL-QOL) questionnaire was developed. The SWAL-QOL is a self-reported tool for measuring the quality of life in patients with oropharyngeal dysphagia.^[6,10]^ A previous study investigated the correlation between dietary stage and quality of life in stroke patients using the SWAL-QOL. It demonstrated that the quality of life was increased by improving dietary stages in patients with dysphagia.^[9]^ SWAL-QOL has also been used to investigate the influence of oral intake on swallowing function and the quality of life.^[11]^ However, there are still insufficient studies on SWAL-QOL outcomes in stroke patients.

Although a fiberoptic endoscopic swallowing study^[12]^ is an important method that has been used recently, videofluoroscopic swallowing analysis remains the standard test for evaluating dysphagia.^[13]^ However, a videofluoroscopic swallowing study (VFSS) has limited ability in predicting the prognosis of dysphagia. The functional dysphagia scale (FDS) reported by Han et al is a useful tool for quantifying and predicting the prognosis of dysphagia.^[14,15]^ Despite its value in explaining the severity of dysphagia, FDS does not predict the long-term prognosis of dysphagia. However, the long-term prognosis of dysphagia is important due to the close association of prolonged dysphagia with lower respiratory tract infections, and high mortality.^[1,16]^ The videofluoroscopic dysphagia scale (VDS) can be used to predict the long-term prognosis of dysphagia patients following stroke.^[17]^ The reliability of VDS based on videofluoroscopic results was shown to be high in a previous study.^[18]^ Presently, VDS can be used to determine the severity of dysphagia with a quantifiable score. It has also been validated for dysphagic patients with various dysphagia etiologies.^[19]^ However, there have been no reports about the relationship between the SWAL-QOL domain and VDS score. Thus, the purpose of this study was to investigate the difference in the quality of life between persons with tube feeding versus oral feeding using the SWAL-QOL. Another purpose of this study was to determine the correlation between the SWAL-QOL questionnaire outcome and VDS scores in stroke patients.

## 2 Materials and methods

### 2.1 Participants

This cross-sectional study was retrospectively conducted with 75 stroke patients with dysphagia symptoms, who were referred to the Department of Rehabilitation Medicine at a tertiary medical center located in Daejeon City in Republic of Korea from January 1, 2017 to March 30, 2019. The exclusion criteria were the inability to provide informed consent, the inability to understand the questionnaire, evidence of symptoms of esophageal dysphagia, and evidence of cognitive disorders such as dementia in the medical history. Seventy-five stroke patients with dysphagia, who could answer the questionnaire, were enrolled. Their mean age was 66.12 ± 11.37 years. Their mean time from onset of stroke was 9.38 ± 14.51 months. All patients had already been assessed by neurologists when they were admitted to the stroke unit. They had undergone computed tomography or magnetic resonance imaging. Fifty-eight patients had cerebral infarction and 17 patients had intracranial hemorrhages. Regarding the location of the brain lesions, lesions were located in the basal ganglia or thalamus in 17 cases, the middle cerebral artery territory in 30 cases, the brain stem area in 16 cases, and multiple territory areas in 12 cases. The patients were classified into the tube feeding group and the oral feeding group. There were 32 patients in the tube feeding group and 43 in the oral feeding group. The general characteristics of each group are described in Table 1. The Ethical Committee of Eulji University, approved the study, which conformed to the tenets of the Declaration of Helsinki. Informed consent was obtained from all participants in this study. All the patients received VFSS and SWAL-QOL questionnaires.

**Table 1:**
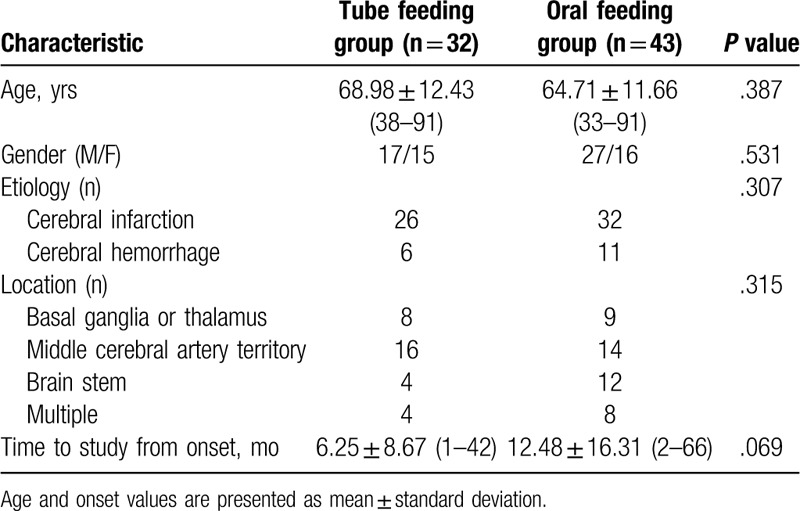
Characteristics of stroke patients with dysphagia (n = 75).

### 2.2 SWAL-QOL questionnaire

The SWAL-QOL (44 items) consists of 10 scales (30 items) and a 14-item dysphagia symptom battery (DSB) for assessing the severity of dysphagia symptoms. The patients were asked to respond to each item based on their experiences during the past month. The 10 scales of the SWAL-QOL are: burden, eating duration, eating desire, food selection, fear, mental health, social functioning, communication, sleep, and fatigue. The first 8 scales are dysphagia specific, whereas the last 2 are generic QoL scales based on principal component analysis by the instrument developers.^[20]^ The responses to each SWAL-QOL item are provided on a 5-point Likert scale. The items within each QoL scale and the DSB were averaged and then linearly transformed to a score of 0 to 100, with lower scores indicating greater impairment.^[20]^ We derived a composite SWAL-QOL score (composite score) following the method of Plowman-Prine et al.^[21]^ It was an average score of the 10 scales, excluding the DSB, which is not considered a scale by the SWAL-QOL developers.^[10,20,22]^ The Korean version of the SWAL-QOL was used in this study. Its reliability and validity has been verified in previous studies.^[23,24]^

### 2.3 VDS for the severity of dysphagia

The VFSS was conducted according to a standard protocol.^[13]^ The procedure was performed by physiatrists in the radiography rooms of the radiology department. The tests were conducted with the patients in the lateral position to display the anatomical structures better. The patients were directed to swallow 2 mL of diluted barium twice. An additional 5 or 10 mL of barium was used according to the investigator's discretion. Subsequently, identical tests were repeated using foods such as yogurt, puddings, rice porridge, and rice with standardized viscosity and quality. The reference diet was pudding. All study procedures were recorded on AVI files (30 frames/s). After all patients finished the VFSS study, the video recordings were collected and each file was given a random number. These files were then copied to 10 DVDs, with each DVD containing all video recordings in a different randomized order. These DVDs were sent to an interpreter for analysis. Two physiatrists analyzed the AVI files. Conclusions were drawn by consensus. For the oral phase, the examiner assigned lip closure, bolus formation, mastication, and tongue-to-palate contact to one of 3 levels: grade 0 = normal, grade 1 = inadequate, and grade 2 = none. Apraxia, tongue thrust, and piecemeal deglutition were evaluated and assigned to one of 4 levels: grade 0 = none, grade 1 = mild, grade 2 = moderate, and grade 3 = severe. The amount of premature bolus loss and the bolus residue in the oral cavity was graded at 4 levels: grade 0 = none, grade 1 ≤10% of bolus, grade 2 = 10% to 50% of bolus, and grade 3 ≥50% of bolus. Oral transit time (OTT) was also measured. In the pharyngeal phase, triggering of pharyngeal swallow, laryngeal elevation, vallecular residue, pyriform sinus residue, coating of the pharyngeal wall, repeated swallowing, and pharyngeal transit time (PTT) were checked. The amounts of vallecular and pyriform sinus residue were classified into 4 levels: grade 0 = no residue, grade 1 ≤10% of the area of the vallecular or pyriform sinus in the 2-dimensional view, grade 2 = 10% to 50% of the area, and grade 3 ≥50% of the area. Aspiration was also checked and graded into 3 levels: grade 1 = no aspiration, grade 2 = supraglottic penetration; and grade 3 = subglottic aspiration. Total VDS score was 100 points, with higher scores indicating more severe dysphagia.^[18,19]^ The VDS consisted of an oral-phase score (subtotal 40 points: the sum of lip closure, bolus formation, mastication, apraxia, tongue-to-palate contact, premature bolus loss, and OTT) and a pharyngeal-phase score (subtotal 60 points: the sum of triggering of pharyngeal swallow, laryngeal elevation, residue in the valleculae, residue in the pyriform sinuses, coating of the pharyngeal wall, PTT, and aspiration).

### 2.4 Statistical analysis

The SPSS ver. 22.0 (SPSS Inc, Chicago, IL) was used for all statistical analyses. The Mann–Whitney *U* test was conducted to compare outcomes of SWAL-QOL between the tube feeding group and the oral feeding group. Statistical analysis by Pearson correlation coefficient was performed to evaluate the correlation between the VDS and SWAL-QOL scores. A 2-tailed *P* value of <.05 was considered statistically significant.

## 3 Results

### 3.1 Differences between the oral feeding group and the tube feeding group

The composite SWAL-QOL score was 48.82 ± 19.51 for the tube feeding group and 53.17 ± 25.42 for the oral feeding group, showing no significant difference between the 2 groups (*P* = .069). The DSB score was 45.69 ± 17.04 for the tube feeding group and 47.28 ± 16.09 for the oral feeding group, showing no significant difference between the 2 groups (*P* = 0.115). The burden score was 43.56 ± 23.67 for the tube feeding group and 59.04 ± 21.42 for the oral feeding group, with a significant difference between the 2 groups (*P* = .005). The sleep score was 45.27 ± 21.94 for the tube feeding group and 67.70 ± 25.89 for the oral feeding group, showing a significant difference between the 2 groups (*P* = .012). The other subdomains of the SWAL-QOL showed no significant differences between the 2 groups (Table 2).

**Table 2:**
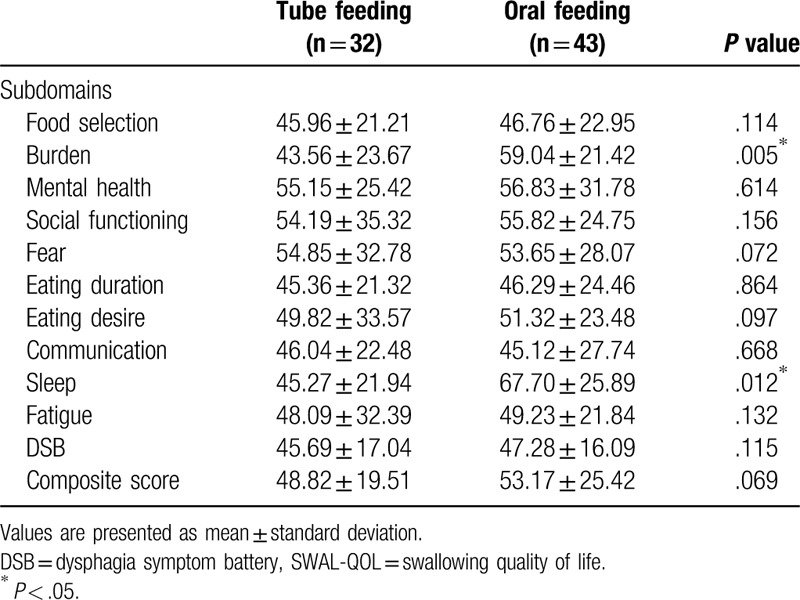
Comparison of SWAL-QOL between oral feeding group and tube feeding group.

### 3.2 Correlation between VDS scores and SWAL-QOL scores

**Figure 1:**
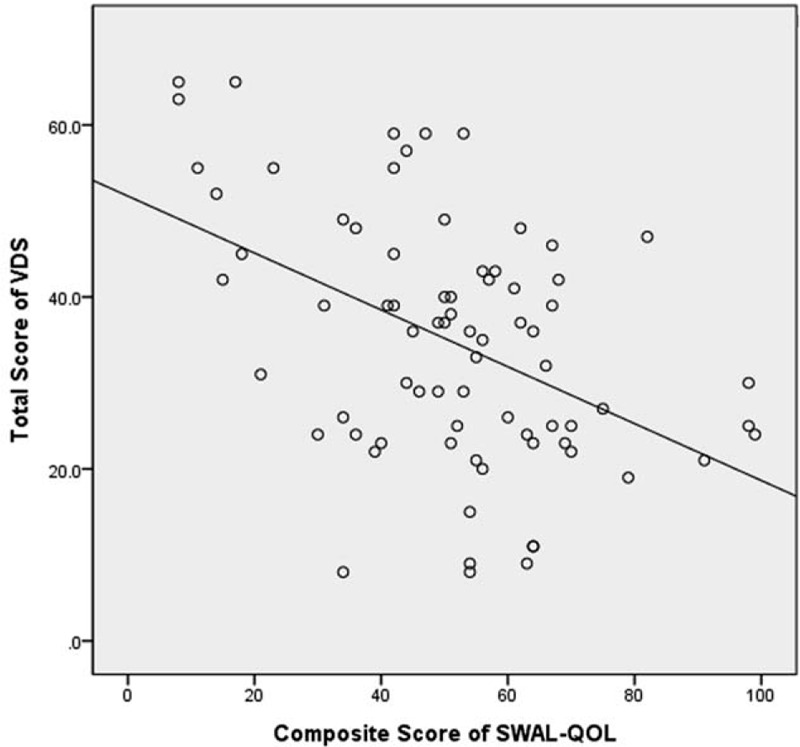
Relationship between total videofluoroscopic dysphagia scale (VDS) score and the composite swallowing quality of life (SWAL-QOL) score.

**Figure 2:**
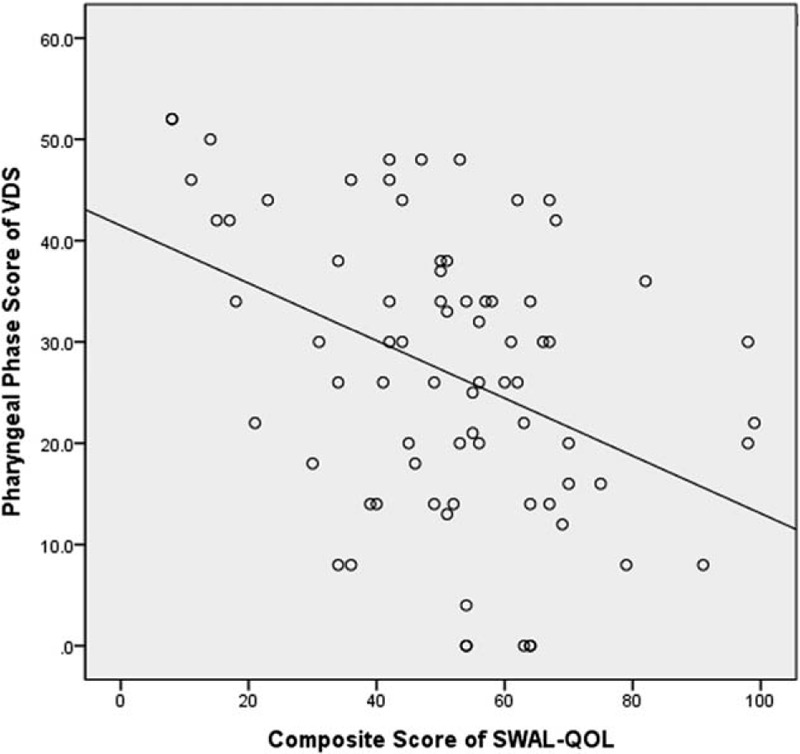
Relationship between the pharyngeal phase score of the videofluoroscopic dysphagia scale (VDS) and the composite swallowing quality of life (SWAL-QOL) score.

**Figure 3:**
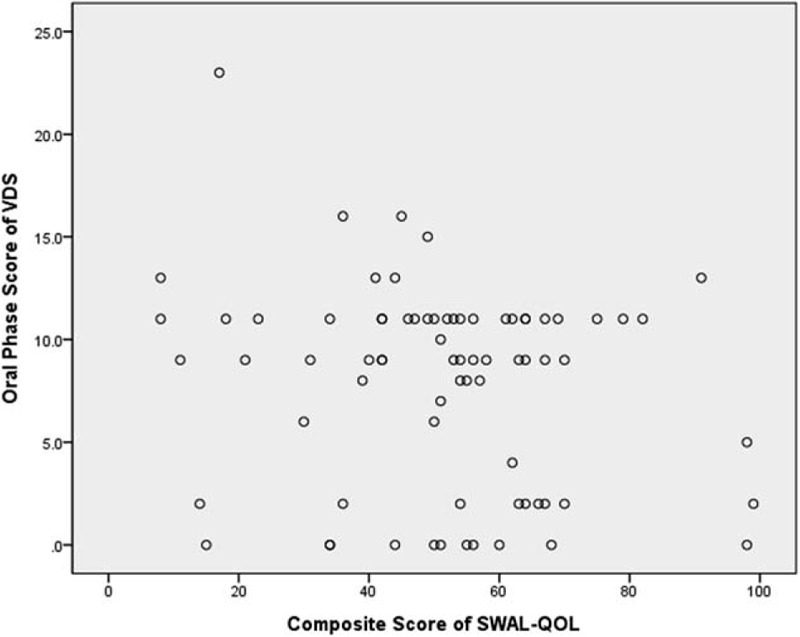
Relationship between the oral phase score of the videofluoroscopic dysphagia scale (VDS) and the composite swallowing quality of life (SWAL-QOL) score.

The total mean VDS score was 35.75 ± 17.43. The composite SWAL-QOL score was 50.97 ± 22.71. There was a significant negative correlation between the composite SWAL-QOL score and the total VDS score (*r* = −0.468, *P* = .012) (Fig. 1). There were also significant negative correlations between the total VDS score and the subdomains of food selection (*r* = −0.295, *P* = .042), burden (*r* = −0.392, *P* = .001), mental health (*r* = −0.362, *P* = .014), social functioning (*r* = −0.370, *P* = .001), and fatigue (*r* = −0.401, *P* = .041). The total VDS score was significantly negatively correlated with the DSB score (*r* = −0.364, *P* = .002) of the SWAL-QOL. There was a significant negative correlation between the composite SWAL-QOL score and the pharyngeal score of the VDS (Fig. 2). The pharyngeal-phase score of the VDS had significant negative correlations with the SWAL-QOL subdomains of burden (*r* = −0.327, *P* = .013), mental health (*r* = −0.348, *P* = .008), and social functioning (*r* = −0.365, *P* = .029). The pharyngeal-phase score of the VDS showed a significant negative correlation with the DSB scores (*r* = −0.359, *P* = .001) of the SWAL-QOL. There was no significant correlation between the composite SWAL-QOL score and the oral-phase score of the VDS (Fig. 3). There was no significant correlation between the oral-phase VDS scores and any subdomain of the SWAL-QOL (*P* > .05). The composite SWAL-QOL score was significantly correlated with the pharyngeal-phase score and the total VDS score (Table 3).

**Table 3:**
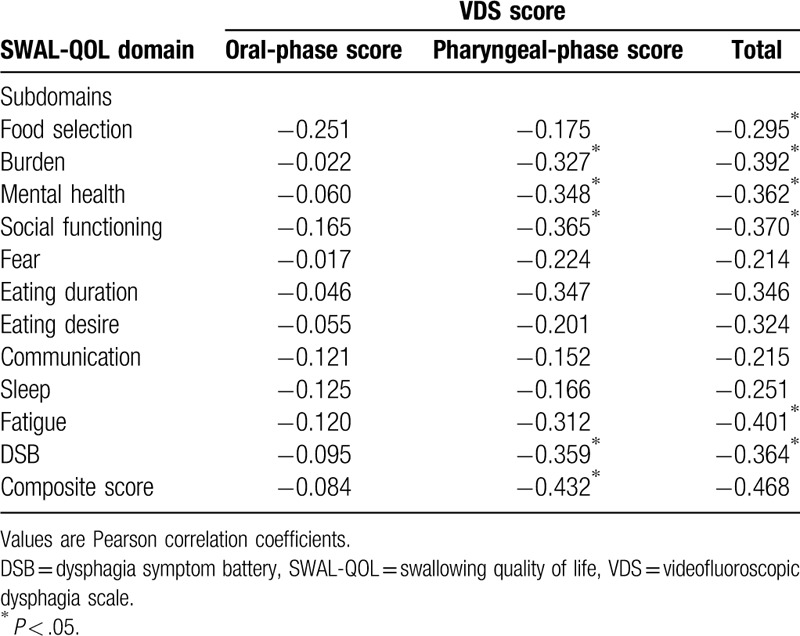
Pearson correlation coefficient between SWAL-QOL outcome and VDS score.

## 4 Discussion

When evaluating dysphagia, most physicians use VFSS or fiberoptic endoscopic evaluation for swallowing function focusing on the physiologic aspect of the symptom. The VDS scale using VFSSs was developed to assess the severity of dysphagia or the effect of dysphagia training.^[8,25,26]^ Until recently, the major focus in the field of oropharyngeal dysphagia has been on physiologic parameters.^[27–29]^ However, swallowing (more broadly, eating) involves not only physiologic food intake, but also social, psychologic, and cultural experiences. Because most clinicians are concerned with only the physiologic swallowing function, there have been insufficient studies on the quality of life of patients with dysphagia. Recognizing this fact, the SWAL-QOL was developed as a disease-specific measurement of QOL in patients with oropharyngeal dysphagia. It has been widely used in effectiveness research as well as in clinical research for dysphagia. The reliability and validity of the SWAL-QOL has been proven in previous publications.^[20,30]^ The validation of Chinese, Dutch, Swedish, Persian, and Korean linguistic versions of the SWAL-QOL have also been proved.^[6,7,23,31,32]^ The Korean version of the SWAL-QOL as a tool to evaluate a patient's quality of life related to dysphagia in stroke patients was used in this study.

A prior study compared SWAL-QOL outcome between a tube feeding group and an oral feeding group in patients with head and neck cancer or neurologic disorders.^[20]^ It showed that the largest differences observed between the tube feeders and oral eaters were in the social functioning and burden domains. Large and statistically significant differences were also observed in the food selection, mental health, fear, and eating desire domains, with the tube feeding group scoring lower than the oral feeding group.^[20]^ Our results are consistent with those of the prior study in the burden subdomain of the SWAL-QOL. However, the sleep domain showed different outcome compared to the prior study. This might be due to different disease entities of the patients. For example, tube maintenance is inconvenient during sleep. Thus, a feeding tube could interfere with the quality of sleep in stroke patients. To improve the quality of life during sleep, we should try removal of the feeding tube as soon as possible. We confirmed the importance of oral feeding in stroke patients with dysphagia.

In 2006, McHorney et al^[10]^ reported correlations between the SWAL-QOL and SWAL-CARE scales with 4 different bolus flow measures in patients with dysphagia of various etiologies. Their study showed that the SWAL-QOL and SWAL-CARE scales were mostly related to OTT and total swallowing duration, while the PTT and penetration-aspiration scale were weakly correlated. The OTT is more likely to reflect disability because the disordered motion of the tongue could increase the amount of time and effort required to move a bolus through the oral cavity. PTT is a measure of automatic activity. It may not sample a portion of the overall swallowing of which the patients are aware. However, PTT alone could not account for the whole severity of pharyngeal swallowing impairment. The pharyngeal score of the VDS more accurately reflects pharyngeal function, such as pharyngeal contraction and motion of the hyoid bone and larynx. Our study showed that the pharyngeal-phase score of the VDS was strongly associated with the total SWAL-QOL score. Based on our results, pharyngeal-phase dysfunction seemed to have more influence on the SWAL-QOL than oral-phase dysfunction. We suggested that it was more important to increase pharyngeal-phase function than oral-phase function, to improve SWAL-QOL of stroke patients. Clinically, dysphagia rehabilitation should focus on the pharyngeal-phase function of swallowing.

This study is a novel report about comparative analysis of subdomains of SWAL-QOL in stroke patients with dysphagia. Furthermore, we established the relationship between SWAL-QOL questionnaire outcome and VDS scores in stroke patients. In our results, the sleep score of SWAL-QOL was lower in tube feeding group than oral feeding group. Clinically, oral feeding is needed instead of tube feeding to improve quality of life during sleep in patients with dysphagia. Swallowing can be divided into 4 phases: oral preparatory phase, oral phase, pharyngeal phase, and esophageal phase.^[27]^ The oral stages of deglutition include mastication, bolus formation, and bolus transfer. Typically, as the bolus passes the anterior faucial arches, pharyngeal swallowing begins. The pharyngeal swallowing response is a rapid, highly coordinated activity that results in velopharyngeal closure, laryngeal elevation and closure, cricopharyngeal relaxation, tongue loading, tongue propulsion, and pharyngeal clearance. In stroke patients with dysphagia, aspiration pneumonia is the leading cause of death following the initial stroke injury. Abnormal epiglottic tilt, delayed pharyngeal phase, and invalid laryngeal elevation belonging to the pharyngeal-phase function were identified as risk factors for aspiration.^[33]^ Therapeutic methods aimed at improving pharyngeal-phase function were important to decrease aspiration and improve SWAL-QOL in stroke patients with dysphagia. In addition, because there were significant correlations between VDS scores and SWAL-QOL outcome, even though they could not be functional eaters after dysphagia rehabilitation, there was the potential for QoL improvement.

Our study has several limitations. First, it was difficult to generalize the results of this study to all stroke patients because it was restricted to a single hospital in South Korea, which might be the reason for discordant results in certain determinants. Additionally, in patients with acute, subacute, and chronic stroke, the degrees of dysphagia and QOL related to swallowing may be different, but subgroup analysis was not performed in this study due to the small number of samples. Further studies supplementing these parameters are needed. Future studies should also include a multicenter study and larger sample size. Second, the number of stroke patients with dysphagia enrolled in the present study were limited, and there were no case-matched control patients, which could potentially create a selection bias. Third, this study was a cross-sectional study, and these findings should be confirmed by long-term prospective studies before establishing a causal relationship between SWAL-QOL outcomes and VDS scores in stroke patients.

## 5 Conclusion

The burden and sleep subdomain scores of the SWAL-QOL in patients in the tube feeding group were significantly lower than those of the oral feeding group. We recommend careful observation and management of physical burdens and sleep problems in tube feeding patients. It is well-known that pharyngeal function plays an important role in swallowing disorders. This study has clinical significance because it is a novel study showing that pharyngeal function can affect the quality of life of patients with dysphagia. Thus, appropriate rehabilitation therapy for pharyngeal-phase dysfunction is needed to improve the quality of life in stroke patients with dysphagia.

## Acknowledgment

The authors thank all participants in this study and also thank HARRISCO and Editage for English language editing services.

## Author contributions

**Conceptualization:** Hyo-Sik Park, Si-Woon Park, Doo-Young Kim, and Jae-Hyung Kim.

**Data curation:** Jae-Hyung Kim and Hyo-Sik Park.

**Formal analysis:** Doo-Young Kim.

**Resources:** Hyo-Sik Park and Doo-Young Kim.

**Supervision:** Si-Woon Park.

**Validation:** Jae-Hyung Kim and Si-Woon Park.

**Writing – original draft:** Doo-Young Kim.

**Writing – review & editing:** Jae-Hyung Kim and Si-Woon Park.
